# Bioengineered Liver Cell Models of Hepatotropic Infections

**DOI:** 10.3390/v13050773

**Published:** 2021-04-27

**Authors:** Francisca Arez, Ana F. Rodrigues, Catarina Brito, Paula M. Alves

**Affiliations:** 1 iBET, Instituto de Biologia Experimental e Tecnológica, Apartado 12, 2781-901 Oeiras, Portugal; farez@ibet.pt (F.A.); anafr@ibet.pt (A.F.R.); anabrito@ibet.pt (C.B.); 2 Instituto de Tecnologia Química e Biológica António Xavier, Universidade Nova de Lisboa, Av. da República, 2780-157 Oeiras, Portugal; 3 The Discoveries Centre for Regenerative and Precision Medicine, Lisbon Campus, Av. da República, 2780-157 Oeiras, Portugal

**Keywords:** liver, hepatocytes, viral hepatitis, *Plasmodium*, hepatotropic pathogens, 3D cell models, in vitro, disease models, bioengineering tools, host–pathogen interactions, drug development

## Abstract

Hepatitis viruses and liver-stage malaria are within the liver infections causing higher morbidity and mortality rates worldwide. The highly restricted tropism of the major human hepatotropic pathogens—namely, the human hepatitis B and C viruses and the *Plasmodium falciparum* and *Plasmodium vivax* parasites—has hampered the development of disease models. These models are crucial for uncovering the molecular mechanisms underlying the biology of infection and governing host–pathogen interaction, as well as for fostering drug development. Bioengineered cell models better recapitulate the human liver microenvironment and extend hepatocyte viability and phenotype in vitro, when compared with conventional two-dimensional cell models. In this article, we review the bioengineering tools employed in the development of hepatic cell models for studying infection, with an emphasis on 3D cell culture strategies, and discuss how those tools contributed to the level of recapitulation attained in the different model layouts. Examples of host–pathogen interactions uncovered by engineered liver models and their usefulness in drug development are also presented. Finally, we address the current bottlenecks, trends, and prospect toward cell models’ reliability, robustness, and reproducibility.

## 1. Introduction

The liver as a critical role in the metabolism and clearance of toxins and pathogens. It is a highly complex organ, composed of cell types of different origins, which during development arise from different germ layers: the liver sinusoidal endothelial cells (LSECs), hepatic stellate cells, and Kupffer cells. These constitute the non-parenchymal compartment of the liver (40%), important for the maintenance of hepatocytes (60%), the parenchymal cells with biosynthetic and detoxification functions [1]. The liver microenvironment induces immune tolerance to antigens presented by non-parenchymal liver cells or expressed by hepatocytes and directly presented on major histocompatibility complex (MHC) molecules. These tolerogenic features favor infection by pathogens that circulate in the blood and specifically target liver cells, establishing chronic infection in hepatocytes [2,3].

Liver infections, which include those elicited by hepatitis viruses and *Plasmodium* parasites, cause substantial morbidity and mortality. Historically, infectious liver diseases have been studied mainly in animal models and hepatic cell lines, focusing on host–pathogen interactions. These models contributed to important knowledge on pathogens’ biology and drug response. Nonetheless, the restricted tropism of these infectious agents and the relevance of liver microenvironment, hepatocyte polarity, and physiological function for host–pathogen interaction have been driving forces for the development of advanced in vitro hepatic infection models.

In this review, we summarize key aspects of hepatic cell models that have been used to study the infection process of major hepatotropic pathogens—namely, hepatitis B and C viruses and the parasite species that contribute the most to human malaria, *Plasmodium falciparum* and *Plasmodium vivax*. We describe important milestones on technical developments of liver modelling and advances on mechanistic knowledge on the biology of infection, as well as contributions for drug development. We further discuss the advantages and drawbacks of these models, the levels of recapitulation of disease, and their suitability for addressing specific scientific questions, as well as their levels of reliability, robustness, and reproducibility.

### 1.1. Hepatitis Viruses

Viral hepatitides are a group of liver diseases caused by one of five known hepatitis viruses: hepatitis A virus (HAV) to hepatitis E virus (HEV) (Table 1). These viruses have similar names and share tropism for hepatocytes. Like any viruses, hepatitis viruses get access to the intracellular environment (entry step), amplify their genetic material (replication step), exploit the cellular machinery to produce viral proteins and assemble into new particles (assembly step), and exit to the extracellular environment (release step). However, hepatitis viruses are very different among each other, which translates into a vast diversity of viral life cycle hallmarks (Table 1). It also leads to a wide spectrum of diseases from mild hepatitis or fulminant liver failure, mainly associated with acute infections, to cirrhosis or hepatocellular carcinoma, more common in chronic infections (Table 1).

Except for the hepatitis C virus (HCV), prophylactic vaccines are available. In 2011, the introduction of direct-acting antivirals (DAAs) represented a significant advance in reducing the global burden of HCV-associated diseases [7]. However, the high error rate of viral genome replication threatens the long-term efficacy of DAAs [8]. In addition, these therapies are very costly for developing nations and challenge the healthcare systems of wealthier countries [9]. Thus, the search for a prophylactic vaccine remains active [10]. From the five hepatitis viruses, hepatitis B virus (HBV) and HCV are the most prevalent, causing major healthcare, social, and economic burden. They also account for the largest share of viral hepatitis-associated mortality (Table 1).

### 1.2. Plasmodium

Malaria is one of the three top infectious diseases worldwide, causing approximately 500,000 deaths yearly [11]. Malaria is caused by parasites of the *genus Plasmodium*. Five *Plasmodium* species are known to infect humans: *P. vivax* (*Pv*), *P. falciparum (Pf)*, *P. ovale (Po)*, *P. malariae*, and *P. knowlesi* [12]. *Plasmodium* infection in the mammalian host initiates in the liver, where each sporozoite traverses several hepatocytes until productively invading one [13]. Inside the invaded hepatocyte, a sporozoite dedifferentiates to trophozoite, which transitions to the liver schizont that undergoes a process of asexual replication and differentiation into blood-stage infectious merozoites. Mature merozoites are subsequently released into the bloodstream and infect red blood cells, leading to malaria-associated pathology [12,14,15]. *Pv* and *Po* trophozoites do not always progress to schizont; instead, they remain in dormant form, the hypnozoites. The latter can remain dormant for months or years until their activation leads to disease relapse [16]. The fact that, during a natural infection, human-infective *Plasmodium* species transit through and mature in the liver makes this step an important target for prophylactic intervention toward malaria eradication. Vaccines that target different stages of the parasite life cycle are under development, but a highly efficacious vaccine is still not available [17]. Therefore, the prevention/treatment of malaria still relies on anti-malarial drugs. The major drawbacks of the available drugs are the wide and increasing resistance of *Plasmodium* [18], and the fact that most therapies fail to target the hypnozoites, which are responsible for malaria relapses. These caveats hamper the eradication of this disease. Therefore, novel drugs with new modes of action that can evade parasite resistance, as well as new drug combinations targeting multiple mechanisms, are imperative for achieving a radical cure for malaria.

## 2. Experimental Models of Liver Infection

Most of the knowledge on infection by hepatotropic pathogens and host response has been generated by employing animal models and classical in vitro two-dimensional (2D) cell models (i.e., monolayer cultures). Due to the restricted host and tissue tropism to mature human hepatocytes of HCV, HBV, and *Plasmodium* species infecting humans, there have been hurdles in the development of infection models, in which not only pathogen entry is recapitulated, but also the complete hepatic life cycle is sustained (replication, assembly, and release for HBV and HCV and parasite differentiation into blood-infectious merozoites for *Plasmodium*).

### 2.1. Animal Models

Chimpanzees have been used as an immunocompetent host when studying HBV and HCV infection and when evaluating novel immunotherapeutic approaches (reviewed by Wieland 2015) [19,20]. Furthermore, initial studies describing *Pv* liver stages were performed on either human or chimpanzee liver biopsies [21,22], and it was in chimpanzee models that the existence of small, non-replicating forms, the hypnozoites, was first reported [21]. However, there are ethical concerns as well as economic constraints related to the use of this animal model [19]. Additionally, for HCV the chronicity rate is substantially lower in chimpanzees than in humans, as well as the severity of infection symptoms, further questioning the use of large primates as study models.

Regarding murine models, their hepatocytes are highly restrictive to HBV, HCV, and human *Plasmodium* infection. Species-specific barriers have been addressed with the expression of human factors known to be required (discussed next), but the biological players of these restrictions are still poorly defined. In that context, recently, Brown et al. (2020) performed murine liver complementary DNA library screening and identified cd302 and cr1l as factors limiting HCV replication in mice hepatocytes [23]. These findings pave the way for the development of next-generation murine models for preclinical testing of HCV vaccine candidates. Moreover, the approach used in this work can be considered for other challenging hepatotropic pathogens, aiding a better understanding of the factors required for their life cycle.

Humanized mouse models have also been established by expressing human hepatic receptors involved in the viral entry in mouse hepatocytes. This approach conferred susceptibility to HBV and HCV entry but resulted in low or no replication [24]. On the other hand, primary human hepatocytes (PHH) engrafted in chimeric mice retained permissiveness to HBV and HCV entry and could sustain viral replication and assembly of infectious viral particles for both cell culture (cc)- and sera-derived particles (reviewed in [25,26]). Hepatic-like cells (HLC) differentiated from human-induced pluripotent stem cells (hiPSC) were also successfully engrafted in mice livers; these cells were shown to be permissive to infection with HCV-positive sera and to support long-term infection of multiple HCV genotypes [27]. Mice engrafted with human hepatocytes are also permissive to *Pf* and *Pv* infection, supporting full sporozoites differentiation into merozoites capable of invading human red blood cells infused into mice [28]. In recent years, these models have also proved to be a valuable source of human hepatocytes for in vitro models, as discussed in Section 2.2.

Immune deficiency is a limitation of these humanized models, currently being addressed with the development of dual-humanized mouse models by engraftment of human hepatocytes and transplantation of human hematopoietic progenitor cells into the bone marrow [25,26,28]. Still, such a model will not completely simulate human immunity since the liver sinusoidal endothelial cells, Kupffer cells and hepatic stellate cells, remain of mouse origin. Furthermore, to engineer faithful in vivo models, an in-depth understanding of the key steps of the pathogen life cycle is required.

### 2.2. D In Vitro Models (Cell Monolayers)

Two-dimensional (2D) cell cultures of hepatic cell lines such as HepG2, Huh7, and HC-04 and their derivatives have been extensively employed in the study of hepatic infection. However, the lack of important hepatic markers and functions in cell lines has long been acknowledged [29], as well as limited permissiveness to challenging hepatotropic pathogens, such as HBV, HCV, and human infectious *Plasmodium*. Genetic engineering of hepatic cell lines expressing specific factors constitutes a validated strategy for establishing a more permissive host cell, as discussed below.

Regarding hepatitis viruses, several cell lines support only some of the steps of the virus life cycle and not necessarily in an orderly manner (e.g., a replication-permissive cell line may not be entry-permissive). This differential permissiveness can be observed by individualizing the different steps of the infection process with the use of recombinant systems. For example, viral particles of non-hepatitis viruses can be pseudotyped with glycoproteins from hepatitis viruses to probe the entry step [30,31], while recombinant inducible systems or RNA replicon systems have long been used to probe the replication steps of HBV and HCV, respectively [32,33]. Additionally, some cell lines enable replication and assembly of viral recombinant particles derived from cell culture production but not the wild-type (*wt*) viruses isolated from patient sera. Indeed, the completion of the entire life cycle of viruses derived from patient sera is commonly used as the ultimate challenge to gauge ‘true’ permissiveness and competence of a cell as host [34]. For *Plasmodium*, there are also reports of cell lines that support *Pf* sporozoite invasion but not development, or just initial development stages but not full maturation into infective merozoites [35,36,37]. Therefore, it is important to contextualize the infection step when referring to concepts of cell permissiveness and competence and, in the case of HCV and HBV, it is important to distinguish between cc and sera viral particles.

Hepatic cell lines played an important role in unveiling specific aspects of the viral infection process, including the identification of host cell receptor molecules involved in virus attachment and entry [38,39,40,41,42,43,44]. Specific clones of the Huh-7 hepatoma cells have been identified as permissive to HCV based on innate immunity defects and upregulation of the hedgehog pathway [45,46], and they are still today the most used cell lines in HCV research. HepaRG is the only hepatocarcinoma cell line naturally susceptible to HBV infection, once differentiated into hepatocyte-like cells [47]. Being a cell line that exhibits innate immunity machinery, it has the potential to unveil innate immune factors involved in antiviral response [48,49].

With the discovery of the specific cell receptor for HBV (Na^+^-taurocholate co-transporting polypeptide, NTCP) [43], the hepatoma cell lines HepG2 and Huh-7 have been modified to express this receptor, becoming susceptible to virus entry [43,50]. HepG2-NTCP and Huh-7-NTCP also sustained viral replication of HBV covalently closed circular DNA (cccDNA) using recombinant production systems, becoming the first in vitro models suitable for studying the complete life cycle of HBVcc particles, although at low levels [43,50]. More recently, it was reported that HepG2-NTCP cells were hardly infected with HBV-positive sera and that a clonal section was required to identify clones producing high titers of infectious progeny [51].

The reasons limiting HCV permissiveness in cell lines are not fully understood, but important aspects have been identified. Limited expression of cell surface receptors, such as CD81 and scavenger receptor class B type I (SRBI), is known to be associated with entry restriction [39,41]. The tight junction membrane proteins claudin-1 and occludin have also been identified as co-receptors of HCV [52,53]. Hepatocyte polarity, essential for the expression and correct localization of tight junction proteins, is poorly recapitulated in hepatic cell lines cultured as 2D monolayer, highlighting the limitations of these models [54,55]. Intracellular factors such as the liver-specific microRNA122 or lipid metabolism gene products are known to be of relevance as well (reviewed in [56]). The transfection of HepG2-CD81 cells with miRNA-122, which facilitates replication of the viral RNA in Huh-7 cells [57], improved RNA replication and infectious virion release, enabling this transgenic cell line to support the entire HCV life cycle for replicon-derived cc particles [58]. With respect to lipid metabolism factors, it is worth noting that until mid-2015, cell culture replication of sera HCV was restricted to two genotypes, when Saeed et al. identified SEC14L2, a cholesterol metabolism gene, as the missing link for pan-genotype replication of HCV primary isolates [59]. Despite the significance of this work, low levels of replication were attained, and the generation of infectious progeny was not reported, suggesting that additional factors are missing.

Studies on *Plasmodium* infection have been limited by the poor access to species infecting humans and the difficulties in maintaining in vitro cultures of *P. vivax*. Therefore, most of the knowledge on the biology of liver-stage *Plasmodium* infection has been generated by employing rodent parasites, such as *Plasmodium berghei* (*Pb*) and *Plasmodium yoelli* (*Py*) [12]. These rodent parasites are amenable to in vitro culture and present a much shorter liver-stage development than the human infectious *Plasmodium* species (48 h versus a minimum of 7 days, respectively) [12,16]. Furthermore, rodent *Plasmodium* species can infect human hepatic cells in vitro, such as the HepG2 and HC-04 hepatoma cell lines, as well as PHH. These models contributed to the identification of host receptors, such as CD81 and SRBI; the processes preceding parasite productive invasion, such as the cell traversal [13,60]; and the study of host–pathogen interactions during liver-stage development, such as host cell remodeling [61]. Nonetheless, the genome differences between human-infective and rodent-infective *Plasmodium* species preclude full translation of the knowledge from rodent to human species. HepG2 and HC-04 cells have been reported to be susceptible to *Pv* and *Pf* invasion, but only HC-04 was reported to sustain *Pf* development [62], and with very poor infection efficiency [36]. Moreover, the proliferative profile of these cell lines hinders their application in the study of human *Plasmodium* species, which present prolonged development times.

The high proliferation, low polarity, and immature phenotype of hepatoma cell lines are surpassed by PHH. However, PHH progressively lose the typical mature phenotype and their very specific physiology and functions in a process known as hepatic dedifferentiation [63]. Strategies employed to delay dedifferentiation of cultured PHH include medium supplementation with hepatic soluble factors or small molecules that modulate specific hepatocyte signaling pathways [64,65], culture over extracellular matrix components, co-culture with non-parenchymal cells, and usage of three dimensional (3D) culture systems (discussed in the next sections). However, the shortage of biological material and high donor variability remains a limitation, highlighting the need for more sustainable and standardized cell sources. Another obstacle particularly evident in *Plasmodium* infection is the high variability in both P*f* and P*v* infection rate between PHH from different donors [35].

Expansion of human hepatocytes (HH) in chimeric mice with humanized livers has been proposed to surpass the limited availability of PHH [66,67]. Ishida et al. reported in vitro infection of HH with HBV patient sera, showing that the model supported the entire HBV life cycle [67]. While promising, this strategy is still technically challenging and not widely accessible. Another alternative to PHH is HLC differentiated from hPSC. In 2012, 2D cultures of HLCs derived from human embryonic stem cell (hESCs) [68] and hiPSCs [69] were shown to support entry and replication of HCVcc. In the same year, Wu et al. (2012) reported the extension of this permissiveness to HCV sera (entry and replication), in addition to permissiveness to HCVcc (entry, replication, and production of infectious progeny) [70]. Furthermore, the authors identified a critical transition point in the differentiation, correlated with induction of the liver-specific microRNA-122, distinguishing non-permissive from permissive cells. These findings were corroborated by Yan et al. (2017) [71]; using HCVcc, the authors identified hepatoblasts as the differentiation stage exhibiting the highest permissiveness and infectivity. hiPSC-derived HLC have also been used to establish infection models for HBV [72,73,74]. Ng et al. (2015) described the infection of hiPSC-derived HLC with the murine parasites *Pb* and *Py*, as well as the human parasites *Pf* and *Pv* [69]. The authors reported invasion and development of all parasite species evaluated, although full development and infectivity toward blood cells has not been assessed. Moreover, development was a less frequent event for *Pf* than for *Pb*. Interestingly, differentiating cells showed high permissiveness to malaria infection at the hepatoblast stage, similar to what has been reported by Yan et al. for HCV [71]. The use of differentiation models offers the advantage of allowing identifying host factors determining permissiveness and essential for each step of the pathogen life cycle. On the other hand, differentiation protocols still suffer from limited reproducibility and scalability. Additionally, drug xenobiotic metabolism is typically immature, closer to fetal hepatocytes than to adult cells, which may limit the applicability of these infection models in drug development. This challenge is being addressed by different strategies, either in vitro, such as media supplementation with small molecules and 3D culture strategies [75,76], or in vivo amplification in chimeric mice with humanized livers [77].

### 2.3. Bioengineered Liver Cell Models

Not all aspects of host–pathogen interactions can be recapitulated in immortalized cell lines or PHH cultured in 2D monolayers. Examples of features that cannot be achieved in conventional 2D cultures are cell polarization, which is required for the correct localization of tight junction proteins that mediate HCV entry in hepatocytes, and long-term cell maintenance, which is required for *Pv* hypnozoite activation after weeks of dormancy. Moreover, the cellular microenvironment plays a key role in determining the gene expression profile, phenotype, and functionality of hepatocytes; interactions with the extracellular matrix (ECM), neighboring cells, and soluble local and systemic cues have been extensively reported as being relevant [78]. Aiming to overcome these limitations, tissue-engineered approaches have been applied to liver cells. The efforts have focused mainly on the development of culture strategies in which clues from the liver microenvironment are recapitulated toward the development of in vitro models that can better address liver diseases.

#### 2.3.1. 2D Cultures

**Figure 1:**
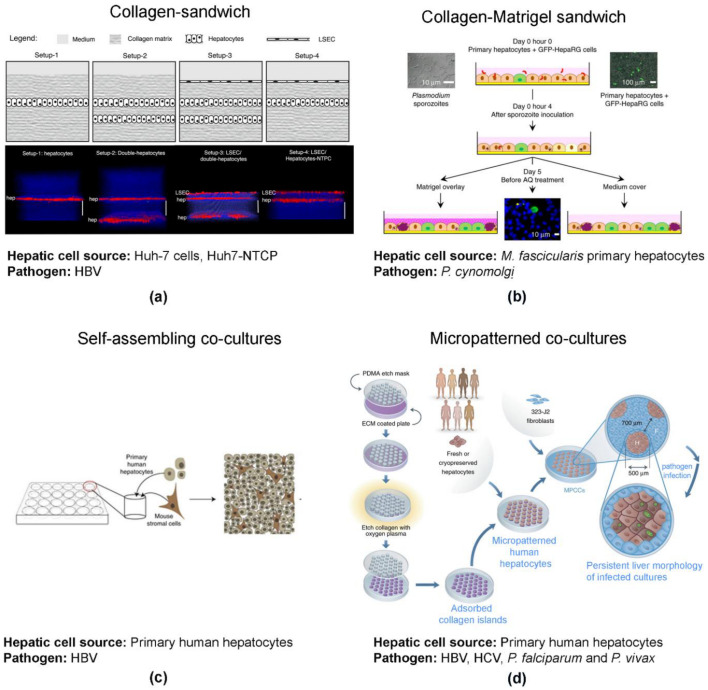
Engineered 2D models for addressing infection by hepatotropic pathogens. (**a**) Collagen-sandwich of a monolayer (setup-1) or double-layer (setup-2) of human hepatic cells without or with (setup 3 and 4, respectively) the presence of a monolayer of liver sinusoidal endothelial cells (LSECs) on top (image reproduced from [79]). (**b**) Monolayer of primary human hepatocytes co-cultured with HepaRG-GFP on top of a collagen-coated surface with a top monolayer of Matrigel^TM^ (image reproduced with permission of [81]). (**c**) Self-assembling co-culture of primary human hepatocytes and non-parenchymal mouse embryonic fibroblast 3T3-J2 cells in a collagen-coated surface (image reproduced from [82]). (**d**) Primary human hepatocytes cultured in islands prefabricated by lithographic methods, surrounded by mouse embryonic fibroblast 3T3-J2 cells (image adapted with permission of [84]).

Engineering tools, such as biomaterials and biofabrication, as well as co-culture strategies, have contributed to improvements in hepatic models based on 2D cell culture. Collagen-based sandwiches and co-culture with non-parenchymal cells have been shown to better reproduce the cell polarity and hepatic functions of primary human hepatocytes and hepatoma cell lines while extending cell viability (Figure 1). This has been achieved via the improvement of cell–cell and cell–ECM interactions [79,80].

Briefly, the collagen sandwich method consists of PHH or hepatic cell lines cultured as monolayers on top of a collagen layer and overlaid with an additional layer of collagen (Figure 1a) [79] or Matrigel (Figure 1b) [81]. In alternative configurations of the sandwich model, PHH are co-cultured with other cell types that can improve hepatocyte function, such as non-parenchymal cells (e.g., liver sinusoidal endothelial cells (LSECs, Figure 1a) [79], liver fibroblasts [80,82], and others, such as HepaRG cells (Figure 1b) [81]. Additional co-culture strategies include self-assembling co-cultures (SACC, Figure 1c) [82,83] and micropatterned co-cultures (MPCCs, Figure 1d). MPCC were pioneered by Bhatia and co-workers [80], resourcing to lithographic processes to fabricate collagen-coated islands in multi-well formats.

Sandwich culture strategies for sustaining the replication of HBVcc and maintaining stable expression of NTCP for 14 days have been described [79]. In SACC cultures, HBVcc replication was sustained up to 30 days and successful co-infection of HBV and HDV has been also reported [47,49]. A sandwich co-culture strategy of simian hepatocytes and HepaRG cells, overlaid with Matrigel sustained hepatic development of *Plasmodium cynomolgi* (*Pc*), a surrogate of *Pv*. The authors reported completion of the full life cycle of the parasite with the development of schizonts, hypnozoites maintenance up to 15 days in culture, and further reactivation of hypnozoites up to 3 weeks of culture [81]. The drawbacks of this model system are the animal origin and batch-to-batch variation of collagen and Matrigel, as well as the chemical gradients between the lower and the top surface of the sandwich.

MPCC, the most explored model platform for liver infections, successfully sustained HBV, HCV, *Pf*, and *Pv* infection with the persistence of viral and parasitic infections for nearly 3 weeks in culture. Although HVCcc infection has been reported, the efficiency was low [85]; for HBV, permissiveness was donor-dependent and several infection readouts suggest that the MPCC system does not yield highly infectious viral particles. Moreover, these are technically challenging cultures. The advantages and disadvantages of these bioengineered 2D cell cultures as infection models have been thoroughly reviewed elsewhere [86,87,88].

#### 2.3.2. Scaffold-Based 3D Cultures

Natural or synthetic scaffolds are commonly employed to mimic physical and chemical clues provided by the tissue ECM governing cellular organization and differentiation. Some of the first tissue-engineered systems proposed for modelling infection by hepatitis viruses were proposed as early as 2001 for HBV [89] and 2009 for HCV [90], employing non-porous scaffolds. These studies report culturing immortalized hepatocytes and hepatoma cell lines (permissive to sera HBV and replicon-derived HCV, respectively) on microcarriers maintained in suspension in agitation-based culture systems, such as a membrane (dialysis) bag [89], or microgravity-based culture systems, such as the NASA rotating wall vessel (RWV), a horizontally rotating cylindrical culture vessel [90]. Microcarriers were developed as a strategy for maintaining anchorage-dependent cells in suspension cultures [91], allowing a more homogeneous distribution of oxygen and nutrients. An improved mass transfer may lead to improved cellular differentiation and culture longevity, overcoming some of the drawbacks of monolayer cultures. Additional advantages of suspension cultures relate to maximizing the cell biomass per culture volume ratio, reducing costs and footprint, and facilitating process scale-up [92,93,94]. In fact, platforms combining microcarriers and suspension culture have been used extensively to enhance the production of HBVcc [95], HCVcc [96], and other viral vectors [97,98,99]. Gong et al. (1998) developed a long-term model of HBV infection employing agitation-based suspension cultures of an adherent immortalized cell line. Replication of HBV derived from patient sera was sustained up to 2 months in culture [89]. Given that HBV chronic infection is characterized by persistence of HBV cccDNA for at least 6 months, a 2-month culture model could be suitable for addressing chronic HBV infection, including the long-term effects on hepatocytes, as well as the emergence of drug resistant strains. More recently, Akahori et al. (2020) employed immortalized hepatic cells cultured in the Cellbed^TM^ scaffold and showed permissiveness to entry, replication, and some level of infectious progeny generation of both sera and cc HBV [100], contrary to HepG2-NTCP cultures that were hardly infected by serum-derived HBV. In 2009, Sainz et al. reported an improved hepatic phenotype of Huh-7 cells cultured in microcarriers in the RWV bioreactor; infection with HCVcc resulted in enhanced genome amplification and production of infectious particles, relative to 2D cultures [90]. These cells adhered to the cytodex microcarriers as multilayers, which promoted agglomeration of 10–20 microcarriers into a 3D structure. These 3D structures were devoid of necrotic centers, a feature commonly reported in 3D culture due to insufficient supply of oxygen and nutrients to the deeper cell layers, and Huh-7 cells presented an enhanced hepatocyte phenotype, characterized by increased expression of phase I and phase II metabolizing enzymes [90]. Huh-7 cells showed physiological localization of the receptors required for the attachment and entry of HCV infection, such as CD81 and SRBI, with prominent detection of the tight junction proteins claudin-1 and occludin and cell polarization consistent with other RWV-cultured cell types [90]. Despite the improvement compared with the monolayer format, the lack of evidence on the formation of bile canaliculi (BC) structures still indicates a deficient cell polarization in this setting [90].

In addition to non-porous microcarriers, other scaffold-based strategies have been employed in the development of 3D hepatic cell models of hepatitis, and more recently of *Plasmodium* infection, such as cell attachment to porous scaffolds (e.g., smart polymers and cellulose sponges) and cell embedding in hydrogels (e.g., Matrigel). Molina-Jimenez et al. (2012) reported a model of HCV infection probed with cc particles based on the culture of Huh-7 cell spheroids within Matrigel™. After 6 days of culture, spheroids showed bile-canalicular structures (BC), demonstrated by apical accumulation of radixin [101], which is reported as a critical requirement for the localization and function of BC transport proteins in hepatocytes [102,103]. The functionality of BC was confirmed by the accumulation of a fluorescent probe specifically transported by the multidrug resistance protein 2 (MRP-2) into these BC structures, which was not observed in monolayers [101]. In the context of HCV infection, the authors reported the apical accumulation of the HCV co-receptors, occludin and claudin-1, surrounding BC as observed in liver biopsies. This hepatocyte-like cell polarity conferred Huh-7 cells permissiveness to HCV entry, although with no difference in replication levels when compared with 2D cultures. The detection of infectious particles in the 3D culture supernatants [101] demonstrated that the model was suitable for studying the entire life cycle of HCVcc. Baktash et al. (2018) applied this platform to Huh7.5 cells and employed it to further understand the multi-stage process of HCVcc attachment and entrance in host cells and the underlying molecular mechanisms [104]. By coupling the polarized hepatic 3D cell model with single-particle imaging, the authors proposed a revised sequence of events involving initial binding to the early entry factors SRBI, CD81, and EGFR at the basolateral membrane, followed by accumulation at the tight junction, associated first with claudin-1 and then with occludin, in an actin-dependent manner. The authors also showed that HCV was internalized via clathrin-mediated endocytosis by an active process requiring EGFR [104].

Despite the wide use of Matrigel™ and other basement membrane mimetic hydrogels, their animal origin and inconsistency between lots makes them less favorable than fully synthetic polymers, such as Mebiolgel [105] and PAG (poly(ethylene glycol)- alginate-gelatine) [106]. Mebiolgel is a synthetic polymer with thermo-reversible gelation, composed of thermo-responsive and hydrophilic polymer blocks. It was employed to generate Huh-7 spheroids, which supported HCVcc replication [105]. The macroporous PAG cryogel, produced from smart polymer responsive to pH and temperature, has recently been employed to cultivate Huh-7.5 spheroids for up to 30 days. These cultures were explored to assess the potential of monoclonal antibodies in blocking the entry of HCVcc [106]. While PEG provides hydrophilicity to the scaffold, the biopolymers alginate and gelatine supply support and adherence moieties, effectively promoting cell–cell contact and cell–ECM interactions underlying the formation of hepatic cell spheroids [106]. Mebiolgel [105] and the PAG cryogel [106] had a modulatory effect on the spheroids, limiting their size to up to 150 µm, while providing enough surface area for cell migration and nutrient and oxygen bulk flow, hindering the formation of necrotic cores. This was crucial for the long-term maintenance of these 3D cultures (up to 63 days) [105]. Furthermore, the non-biological origin of the constituents of these platforms makes them advantageous over Matrigel-based models specifically for addressing host responses to HCV infection and host response to anti-viral drugs, which might be affected by the animal-derived factors present in Matrigel.

Nugraha et al. (2011) proposed the use of galactosylated cellulosic sponge for generating a hepatic model, with the advantage of reduced drug absorbency when compared with other scaffolds, such as collagen [107]. Ananthanarayanan et. al. (2014) employed cellulosic sponges as an alternative to the previously described scaffolds to establish hepatic spheroids of Huh-7.5 cells, resulting in higher levels of entry and replication of HCVcc, [108]. The 1 mm thick sponges can be produced in bulk and their small dimension reduces drug absorption [107]. As reviewed by Wells (2008), hepatocytes maintain a differentiated phenotype in soft supports and dedifferentiate on stiff supports [109]. The macroporous and soft nature of the cellulosic sponge provided control over spheroid size and the ideal rigidity to prevent cell spreading maintaining hepatocytes differentiation [66,67]. Assessing the elastic modulus of the sponge demonstrated its resemblance to the native human liver [107]. Furthermore, the conjugation of the cellulose with galactose ligands, which interact weakly with asialoglycoprotein receptors (ASGPR) on the hepatocyte plasma membrane [107], provided chemical cues to the cells to reorganize into spheroids [66,67]. The combination of the physical and chemical cues contributed to the generation of spheroids of polarized PHH and Huh 7.5 cells, exhibiting defined apical and basolateral domains, as demonstrated by the spatial segregation in the cell membrane of MRP-2 (apical) and CD147 (basolateral). Additionally, CD81 was localized in the basolateral domain and SRBI, claudin-1, and occludin in the tight junctions associated with the apical side. However, Huh-7.5 did not form BC-like structures in this system [108]. Regarding HCV infection, the authors showed that spheroids from both cell sources could mediate the entry of pseudoparticles harboring HCV envelope glycoproteins, however with different permissiveness; higher intracellular levels of HCV were detected in Huh 7.5 cells than in PHH, and when infected with the HCV live strain JFH-1, only Huh 7.5 spheroids could sustain viral replication, consistent with previous reports in other culture formats [85,110]. Finally, the authors performed a proof-of-concept of the model applicability in drug discovery by challenging the cultures with an anti-CD81 antibody, which neutralized the HCV infection in a dose-dependent manner [108]. Importantly, the authors not only showed the suitability of the platform for preclinical drug development targeting HCV infection but also the flexibility of the developed platform for application to different cell sources [108].

**Figure 2:**
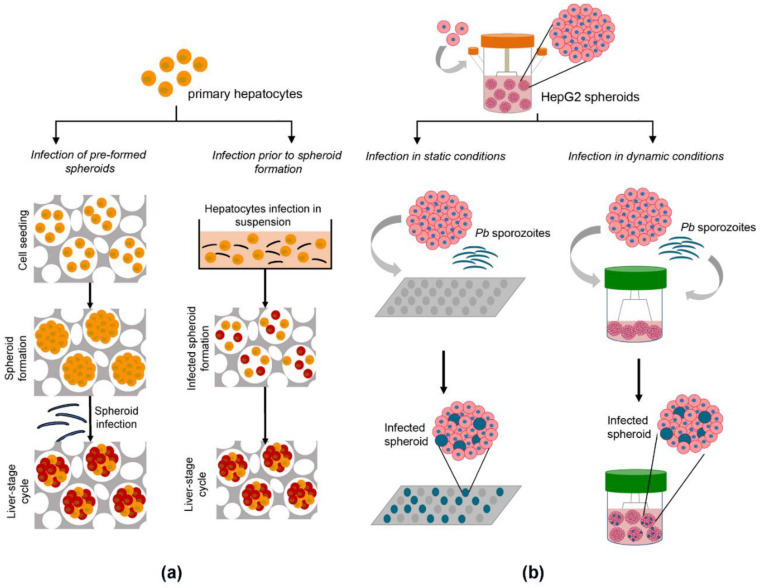
Engineered 3D models for addressing *Plasmodium* infection. (**a**) Spheroid infection with *P. cynomolgi* (*Pc*) in cellulosic sponge scaffolds. Spheroids of primary simian hepatocytes generated in cellulosic sponges and infected with *Pc* sustained *Pc* invasion and development (left panel); single cells were infected with *Pc* sporozoites before spheroid formation and infected cultures were seeded in the cellulosic sponges for the generation of *Pc*-infected spheroids (right panel). Image adapted with permission of [111]. (**b**) Scaffold-free spheroid infection with *P. berghei* (*Pb*). Spheroids generated in stirred-tank culture systems were transferred into 96-well plates (static, left panel) or into smaller scale stirred-tank culture systems (dynamic, right panel) and infected with *Pb* sporozoites, sustaining *Pb* invasion and development [82].

Employing the same cellulosic sponge-based strategy, Chua et al. (2019) published the first evidence of *Plasmodium* infection in a hepatic 3D model (Figure 2a) [111]. The authors hypothesized that hepatocyte maturation was critical for sustaining the full life cycle of *Pc* and *Pv*, including latent hypnozoite forms. The cellulosic sponge platform was implemented employing simian hepatocytes, again demonstrating the flexibility of this system for use with a range of hepatic cell sources. Simian hepatocytes were plated prior to or following *Plasmodium* infection (Figure 2a), with the formation of hepatic spheroids that maintained structural integrity up to 60 days when not infected and up to 30 days when infected by *Pc*.

Hepatic urea synthesis was employed as a surrogate of hepatocyte functionality, being detected in simian spheroids at higher levels than in hepatocyte monolayers and sustained for 22 days of culture [111]. Despite that, there was a clear decline in urea production even in the spheroids after the first 8 days of culture. The conditions optimized for *Plasmodium* infection included a sporozoite to hepatocyte ratio of 2:1, the ratio leading to a higher infection rate while minimizing the risks of contamination derived from sporozoites of non-sterile mosquitoes. Hepatocytes exposed to the parasites before 3D culture had a 4-fold higher infection rate than hepatocytes infected as spheroids embedded in the cellulosic sponge [111], possibly due to barrier limitations of the scaffold. The authors described the exoerythrocytic forms (EEFs) as punctuated parasites, and these were detected throughout the entire spheroid structure. Developing schizonts, from primary infection and reactivated hypnozoites, fully matured into infectious merozoites capable of infecting simian erythrocytes. As a proof-of-concept of the application of this system in drug assays, spheroid cultures were incubated with anti-plasmodial compounds, such as the standards of care atovaquone (targeting liver-stage schizonts but not hypnozoites) and primaquine (targets both liver-stage schizonts and hypnozoites), as well as KDU691 (an exploratory drug with potential anti-relapse effects, previously reported to achieve radical cure in 2D monolayers but not in vivo). The assay readout was the detection of *Pc*-HSP70 protein by immunofluorescence microscopy. Although this is not a convenient analytic method for high-throughput drug screening applications, the authors could detect the distinct anti-plasmodial effects of primaquine (near-complete infection clearance) and atovaquone (solely schizonts clearance) but did not observe complete elimination with KD691, consistent with in vivo data [112]. The authors extended the impact of the model by demonstrating its applicability to PHH and the feasibility of infecting PHH spheroids with *Pv*. Overall, this was the first report that spheroid-based cultures support the invasion and complete liver-stage development of *Pc* parasites in a system that could be employed for anti-plasmodial drug screening.

Decellularized liver tissues have been extensively proposed as scaffolds for tissue grafts and whole organ engineering. These scaffolds provide the actual tissue architecture and extracellular matrix of liver tissues and also have the potential for in vitro modelling of liver diseases, as reviewed by McCrary et al. (2020) [113]. The first study employing native scaffolds for hepatitis infection models has recently been published. Zhang et al. (2019) reported the successful engraftment of PHH and HepG2-NTCP in decellularized human livers and HBVcc infection, showing the robustness of the system with different cell sources [114]. Moreover, the authors demonstrated its potential for drug discovery applications employing the anti-viral Entecavir. Importantly, it was shown that the hepatocyte phenotype and HBV infection and replication was similar in scaffolds derived from healthy and cirrhotic patients, supporting the use of decellularized cirrhotic tissue as surrogates and minimizing the bottleneck of limited access to healthy livers. With this work, Zhang et al. (2019) contributed to the increase of the short repertoire of 3D in vitro models sustaining HBV infection.

Overall, these studies show scaffolds as potent tools in the generation of 3D hepatic cultures. Among the different scaffolds, the cellulosic sponge presents high flexibility; it is applicable to several cell sources and capable of sustaining the full life cycle of several pathogens. Moreover, it has been shown to be better suited for drug discovery applications due to minimized drug retention. Nevertheless, this scaffold still shows some extent of drug retention [107] and poses a barrier to pathogen invasion, leading to lower infection rates [111]. Moreover, it is not compatible with high content imaging [111], a tool widely used to characterize 3D in vitro models and to assess drug efficacy in phenotypic screenings [35]. 

#### 2.3.3. Scaffold-Free 3D Cultures

Several methodologies have been proposed for the generation of cell spheroids without the addition of scaffolds and exogenous matrix components, such as microgravity, gravity (on hanging drop or low-adherence surfaces), and agitation (orbital shaking or stirred-tanks systems). A comparative study between PHH spheroids produced in ultra-low adherence plates and collagen–Matrigel sandwich cultures showed that hepatocyte in spheroids presented high metabolic performance and synthetic functions and were stable for 2 weeks, whereas PHH in sandwich format lost hepatic functionality within that same period [115]. Furthermore, the same team showed the suitability of PHH spheroids for modelling drug-induced liver injury, steatosis, and cholestasis and for mimicking viral infection by viral transduction with recombinant adenovirus [116].

Our team has shown that culture of PHH as spheroids in stirred-tank bioreactors in perfusion modes extended culture time and sustained high gene expression of phase I and phase II metabolizing enzymes for at least 4 weeks, with induction by prototypic drugs [117]. The model could be improved by co-culture with mesenchymal stem cells, leading to increased hepatocyte polarization, functionality, and xenobiotic activity, with less inter-donor variability [118]. Stirred tank-based systems were also applied to hepatic cell lines, such as HepaRG and HepG2 [119,120,121], which could be maintained in culture up to 7 weeks with xenobiotic activity assessed by the detection of the intermediate compounds derived from the activity of the specific CYP450 isoforms and by exposure to acetaminophen (APAP) [119]. Taking advantage of stirred tank culture systems, our group established the production of spheroids of HepG2 and HC-04 cells and their infection with *Pb* [121]. This work presents the first scaffold-free spheroid model sustaining the complete liver stage of *Plasmodium.* Moreover, the cell lines employed have been described as sustaining human *Plasmodium* infection (e.g., *Pv* and *Pf*, respectively) [62,122], which is expected to facilitate the translation of the platform to human parasites. Importantly, we have shown the predictive value of the platform for anti-plasmodial drug discovery. The anti-plasmodial activity of both atovaquone (standard of care) and M5717 (drug candidate) was equivalent to what has been observed in vivo [121]. Contrary to what has been described, we performed infection on pre-formed spheroids. Furthermore, we could implement two platforms for infection of hepatic spheroids and drug interrogation: (1) infection and drug challenge in stirred tank cultures (dynamic, Figure 2b, right panel), useful to study the time-response (kinetics) of the drug activity, and (2) infection in medium-throughput platforms (static), such as 96-well plates (Figure 2b left panel), useful to study dose-responses. Furthermore, the stirred tank-based platform is scalable and can also feed medium- to high-throughput screenings. Currently, this platform is being translated to human primary human hepatocytes for the employment of this system in *Pf* infection. The flexibility of the dynamic platform in terms of cell source and prolonged culture times makes it suitable for addressing *Pv* hypnozoites’ biology, potentially contributing to an enlargement of the limited pipeline of anti-plasmodial drugs that target hypnozoites and overcome *Plasmodium* drug resistance.

#### 2.3.4. 3D Co-Cultures

One of the few 3D co-culture liver cell models proposed so far has been employed to study hepatic response to both sera and cc HBV infection [123]. In this microfluidics system, PHH were co-cultured with Kupffer cells in collagen-coated polystyrene scaffolds, with recirculation of culture media at a constant flow rate. Both in HBV-infected PHH monocultures and co-cultures with primary Kupffer cells, no pro-inflammatory response or induction of hepatic interferon beta (IFN-β) and interferon-stimulated genes (ISG) signaling pathways were detected. Nevertheless, alterations in the cytokine and chemokine fingerprint of HBV-infected cultures were observed, with a good correlation with those observed in HBV-infected patients. Despite the lack of pro-inflammatory response to HBV infection in Kupffer cell–PHH co-cultures, exogenous activation of the Kupffer cell population with lipopolysaccharide (LPS) initiated a pro-inflammatory response that was stronger in HBV-infected than non-infected cultures, leading to inhibition of viral replication [123]. This suggests that the 3D PHH–Kupffer cell co-cultures can be employed to dissect host–pathogen interaction and the generation of antiviral responses against HBV from specific cell types.

A few scaffold-free spheroid co-culture models have also been developed for drug toxicity applications. Rebelo et al. (2017) reported the establishment of human hepatic spheroids in stirred-tank bioreactors composed of PHH and bone marrow-derived mesenchymal stem cells (BM-MSC) [118], shown to support liver function and to have a therapeutic effect in acute liver failure and liver fibrosis [124]. In this system, a spatial arrangement of the cells has been reported, with BM-MSC becoming restricted to the outer layers of the spheroids with no penetration into the hepatic core structure, mimicking the architecture of hepatic stellate cells and hepatocytes in the liver tissue. PHH in co-cultures showed improved hepatic functionality and metabolic stability, potentially driven by increased deposition of ECM and IL-6 expression (exclusive of co-cultures), an interleukin that directly promotes hepatic biosynthetic functions [125]. Bell et al. (2016) increased the complexity of PHH spheroids by co-culturing PHH with a mixture of NPCs [116]. The authors reported NPC migration into spheroids, and the functionality of all cellular components was confirmed by directed stimuli-LPS for Kupffer cells and transforming growth factor-beta (TGF-β) for hepatic stellate cells [126]. Both co-culture strategies targeted APAP toxicity, with the demonstration of its metabolism into the toxic intermediate [118,126]. NPC were shown to have a protective effect, with a potential role of micro-RNA related mechanisms [126]. Overall, these studies demonstrate the potential of 3D co-culture models for studying disease progression and for assessing drugs.

Recently, organoids emerged as a recapitulative model to study hepatic physiology and pathophysiology. Despite being a term generalized among the scientific community for many decades [127], with the seminal work of Hans Cleavers group and the developments in the area thereafter, the organoid definition has been updated to a 3D structure in which cells spontaneously self-organize into progenitors and differentiated functional cell types that resemble the original organ and recapitulate some of its functions [128,129]. So far, liver organoids have not been employed to study infectious diseases. Up to now, there is only one report on the use of liver 3D cell models derived from human stem cells to model infectious diseases, specifically HBVcc infection [130]. The authors employed hiPSC-derived endoderm cells, which can be obtained with patient-specific genetic background. The liver models were established by co-culturing hiPSC-derived endoderm cells with HUVECs and bone-marrow mesenchymal stem cells in microwell plates for 3D cell culture in a chemically defined differentiation medium. The authors reported enhanced hepatic differentiation (hepatic specific morphology and structural organization, function, and gene expression) up to 20 days of culture compared with hiPSC-derived HLC from the same genetic background. The authors also showed that the presence of the microenvironment provided by the mesenchymal and endothelial cells was a determinant for hepatic differentiation, with an earlier and more robust expression of hepatic-specific genes relative to classically differentiated hiPSC-HLC [130]. These observations corroborate previous reports defining the microenvironmental key elements essential to trigger hepatic differentiation during liver development [131,132]. The generated 3D co-cultures were infected by HBVcc, sustaining virus replication and propagation in levels comparable with those of PHH at 10 days post-infection (dpi). hiPSC-HLC cultured in 3D presented higher susceptibility to HBV infection than hiPSC-HLC differentiated in 2D cultures, despite the comparable levels of NTCP in both cell types, suggesting that in addition to NTCP other factors are important for virus infection, probably related to the improved differentiation/maturation state. The extended time of the 3D co-cultures allowed viral replication up to 20 dpi, the time at which the viral progeny was harvested. Collected viruses were capable of infecting hepatocytes isolated from humanized mice, in which HBV could complete its life cycle and produce viral particles [130]. Increased viral load led to increased inhibition of hepatic-specific gene expression in the 3D model, which was also observed in PHH monocultures. Additionally, these 3D co-cultures reproduced liver dysfunction caused by HBV viral infection—observed by increased expression of transaminases ALT and AST and lactate dehydrogenase (LDH), markers of early acute liver failure—together with alterations in cellular ultrastructure. The expression of epithelial-to-mesenchymal transition (EMT) markers was significantly upregulated by HBV infection. Finally, to depict the host-immune response to HBV infection, infected 3D co-cultures were exposed to exogenous interferon-alpha (IFNα). This induced the expression of antiviral genes, resulting in the inhibition of viral replication but also in the inhibition of hepatocyte gene expression levels, suggesting that the innate immune activation could effectively inhibit virus replication but would induce additional hepatic injury. Overall, this hiPSC-derived 3D co-culture model reproduced the host response to HBV infection—namely, the virus-induced liver injury observed in immunosuppressive patients [133], the host innate immune response [134], and the HBV involvement in driving EMT associated with liver cancer development [135]. This model tackles several of the current bottlenecks being derived in a chemically defined medium without the interference of Matrigel^TM^ and sourcing to hiPSC, an unlimited cell source. Moreover, since the host genetic background might play an important role in virus-induced outcomes, the fact that hepatic cells can be produced from hiPSC, which can be derived from different genetic backgrounds, is a promising approach for addressing the contribution of the host background for virus–host interactions, as well as for the development of precision medicine approaches. The model may be further improved by substituting HUVEC cells with a more physiological endothelial cell source and, ideally, all cell types derived from the same genetic background.

## 3. Current Challenges, Trends and Future Directions

In vitro liver models are growing toward a better representation of human tissue, providing more accurate knowledge on host–pathogen interaction and drug response. Nonetheless, there is still a gap in the representation of the liver cellular complexity and architecture. Increasing evidence points to an important role of non-parenchymal and immune cells in the liver response to infection by HCV, HBV, and *Plasmodium*. Analysis of liver biopsies of HCV-infected patients revealed that Kupffer cells secrete IFN-β, playing a role in spontaneous HCV clearance and suggesting a role for plasmacytoid dendritic cells (pDCs) in the intrahepatic IFN response [136]. Still, HCV may escape from the liver innate response by blocking IFN-β and ISG induction through a process not totally understood [136]. Therefore, knowledge of the activation of the host innate immunity and the interplay between host and HCV are essential for understanding the establishment of chronic HCV infection. In that context, a recent model employing stem cell-derived HLC has been described for studying chronic HCV infection based on blockade of JAK/STAT signaling [137]. Contrary to HCV, HBV clearance is delayed by the deficient DNA-sensing machinery of hepatocytes [123,138]. However, this virus activates macrophages when in high titers, inducing a response mainly through the production of inflammatory cytokines such as TNF-α and IL-6, which may play a role in limiting HBV infection and mediating viral clearance [138]. Kupffer cells have been reported to constitute the entry gate of *Plasmodium* into hepatocytes [139], but the initiation of an immune response during this liver-stage of the infection is not known. Nonetheless, effective CD8+ T cell migration and surrounding of infected hepatocytes occurs, although not leading to a complete infection clearance [140]. Few to nearly none of the in vitro models described so far represent the complexity of this cellular interplay for the study of hepatotropic pathogens. Furthermore, drugs can activate the hepatic immune system and Kupffer cells, contributing to drug-induced liver injury, one of the most common causes for drug withdrawal from the market. The lack of cell sources that can provide sufficient numbers of functional cells has been one of the bottlenecks of liver cell modelling. This will potentially be alleviated by alternatives, such as human stem cell-derived hepatocytes and NPCs, once the fetal type and poor functional maturation issues are solved [75]. Another alternative is the use of genetically engineered primary cells, which have been shown to allow expansion of donor and tissue-specific cell cultures [59], as well as to modulate liver functional heterogeneity in human hepatocytes [141] and in vivo expanded primary hepatocytes [67].

Advanced in vitro models contemplating heterogeneous cellular components hold the potential to improve drug discovery and development by better mimicking pharmacokinetics and pharmacodynamics, as well as understanding mechanisms of disease progression and host response in vitro. In recent years there has been a rapid expansion of 3D cell models that address the liver microenvironment, including the liver architecture, the cell–ECM interplay, and the liver heterotypic cellular components. As highlighted in this review, spheroids, organoids, and decellularized matrices constitute some of the widely used strategies for 3D liver modelling to recapitulate such features. Bioengineering tools such as bioreactors, microfluidics, biomaterial scaffolds and 3D bioprinting can facilitate the recapitulation of the complex spatial patterning of liver cells, their reciprocal crosstalk, and the communication with the circulatory system. In the last decades, bioreactor technology has been employed for the long-term maintenance of 3D liver cultures [117,142]. Longevity is a crucial feature for addressing prolonged and recurrent infection and the liver pathology induced by the infectious agents, the therapeutic effect, and the toxicity of repeated drug administration [117,118,121]. Stirred-tank bioreactors allow non-destructive sampling throughout the culture time, which is particularly useful for relapse studies of *Plasmodium* infection [81] or persistent viral infection. Culture of PHH and hepatoma cell lines in perfusion stirred-tank bioreactors promotes spheroid formation and assures the optimal concentration of nutrients and soluble factors and the gradual removal of metabolic by-products by automated perfusion, whilst offering online monitoring and control of critical parameters, such as pH and partial oxygen pressure (pO_2_) [91]. Our team has shown that not only hepatocyte spheroid maintenance but also *Plasmodium* infection and viral vector transduction can be accomplished in stirred conditions [121,143], opening the possibility for large scale production of cell models of hepatic infection, given that stirred-tank bioreactors are easily scaled-up.

On the other hand, miniaturization and parallelization can be achieved by microfluidic-based approaches, such as organ-on-a-chip. These are based on cutting-edge microfabrication technologies that culture functional units of living human organs in an integrated system of channels and chambers with the manipulation of fluids. These platforms allow presenting a variety of extracellular cues to cultured cells with spatiotemporal precision [144]. Liver chips have been used in different formats, including the culture of hepatic spheroids and organoids with the effect of flow [145]. Hepatocytes can be cultured in settings with mass transport properties similar to the liver acinus, or in co-culture with other cell types to reproduce the cell–cell paracrine interactions [146]. One of the liver mimetic features more often described for these platforms is hepatocytes zonation. This is a crucial feature for accurately mimicking the liver function, as liver zonation determines hepatocytes metabolic functions [147]. Furthermore, the metabolic heterogeneity of hepatocytes may result in zone-specific drug toxicities (reviewed by Cox et al. 2020 [148]); it is important to employ these models for drug discovery purposes. The flow modulation can be coupled with micropatterning techniques to define cellular organization [149,150]. Organ-on-a-chip approaches are promising for the creation of interactive co-culture environments where different tissues can be connected through the microchannels, potentially including an immune system [151,152], which would be particularly useful in studying the role of immune response in infectious liver diseases, such as chronic viral hepatitis.

Emerging technology is 3D bioprinting, a bottom-up strategy for reconstructing tissue-like structures through simultaneous deposition of bioinks (biomaterial-based matrices) and living cells, precisely positioned to form 3D complex structures. The compatibility of 3D bioprinting with bioreactor and microfluidic systems [153] brings 3D spatial resolution to precisely define the architecture of the cell model. A 3D bioprinted spheroid microfluidic model showed the maintenance of HepG2 spheroids up to 30 days in culture maintaining functionality and demonstrating susceptibility to APAP hepatotoxicity reported in other animal and in vitro models [153]. New approaches of 3D bioprinted livers include co-cultures of PHH, hepatic stellate cells, and endothelial cells [154,155]. Although not yet optimized, the use of decellularized ECM-based bioinks is also emerging for the fabrication of tissues and organs with the unique biological properties of the liver. However, decellularization procedures still need to be improved so as not to damage the ECM structure, and there is also the issue of batch-to-batch variations due to compositional differences between distinct sources [156].

Advanced in vitro liver models are greatly improving in complexity and mimetic potential in what concerns hepatic function, infection, and modelling of other diseases, as well as drug response. Nevertheless, each model presents advantages and drawbacks to be considered depending on the application. Merging the distinct liver microenvironment components into advanced hepatic cell models has the potential to revolutionize our understanding of host–pathogen interactions, host-driven responses, and disease progression. It is also expected to greatly impact the development of novel therapeutics targeting recently uncovered mechanisms for overcoming drug resistance and hepatotoxicity effects unpredicted in the currently employed preclinical models.

## Figures and Tables

**Table 1:** Overview of hepatitis viruses ^a^.

	Family, Genus	Attachment and Entry Factors/Receptors	Main Life Cycle Hallmarks	Associated Diseases	Prevalence and Mortality ^b^
	Genome				
	Genotypes				
**HAV**	*Picornaviridae*, *Hepatovirus*	Viral: • - Host: • HAVCR1	• Acute infection • Exists in two envelopment forms: naked and quasi-enveloped [4] • Does not integrate into the host genome • Direct translation of viral genome (IRES-mediated) • Release by cell lysis	• Mild hepatitis (common) • Fulminant acute hepatitis (rarely)	Every year, 1.4 million people are infected, and in 2016 7134 deaths occurred, mainly due to fulminant hepatitis.
	Linear ssRNA (+)				
	6 (I to VI)				
**HBV**	*Hepadnaviridae*, *Orthohepadnavirus*	Viral: • (Large) S Host: • NTCP • HS	• Acute infection, may evolve to chronic • Enveloped virus • Can integrate into the host genome • The genome is a mini-chromosome transcribed by host polymerases into 4 mRNAs • Release by budding (ESCRT-dependent)	• Hepatitis • HCC • Cirrhosis	In 2015, 257 million people were estimated to be chronically infected, and 887,000 deaths occurred, mostly from cirrhosis and HCC.
	Circular dsDNA (c)				
	10 (A to J)				
**HCV**	*Flaviridae*, *Hepacivirus*	Viral: • E1/E2 Host: • CD81 • HS • LDLR • SRBI • CLDN1 • OCLN • NPC1L1	• Chronic infection • Enveloped virus • Does not integrate into the host genome • Direct translation of the viral genome (IRES-mediated) • Release by exocytosis after budding into the ER via lipoprotein assembly and secretion (reviewed in [5])	• Hepatitis • HCC	In 2016, 71 million people were estimated to be chronically infected, and 399,000 deaths occurred, mostly from cirrhosis and HCC.
	Linear ssRNA (+)				
	6 (1 to 6)				
**HDV**	*Unassigned*, *Deltavirus*	Viral: • HBV’s (Large) S Host: • NTCP • HS	• Acute infection • Enveloped virus • Does not integrate into the host genome • Satellite virus: infection requires the host cell to be co-infected with HBV. • Replication occurs by rolling circle, single genome being cleaved/ligated by HDV ribozymes • Release by budding using HBV proteins	• Fulminant acute hepatitis • Severe chronic active hepatitis • HCC	Affects 5% of people with HBV; deaths included in the HBV mortality.
	Circular ssRNA (-)				
	8 (1 and 8)				
**HEV**	*Hepeviridae*, *Orthohepevirus*	Viral: • - Host: • HS • Unknown receptor	• Acute infection, may evolve to chronic • Exists in two envelopment forms: naked (enteric route) and quasi-enveloped (bloodstream) • Does not integrate into the host genome • Direct translation of the viral genome (caped genome) • Released by budding (ESCRT-dependent) at both the basolateral and apical side of the hepatocyte. The latter leads to striping-off of the envelope by bile acids resulting in naked particles [6]	• Mild hepatitis • Associated with a high mortality rate during pregnancy (fulminant liver failure)	Every year, 30 million people are infected with 3.3 million symptomatic cases, and in 2015, 44,000 deaths occurred, mainly due to fulminant hepatitis during pregnancy.
	linear ssRNA (+)				
	4 (1 to 4)				

^a^ If not otherwise indicated, the content source of this table was obtained from the Viral Zone website (viralzone.expasy.org). ^b^ Source: WHO data and factsheets on hepatitis viruses. CLDN1: claudin 1. HCC: Hepatocellular carcinoma. HS: Heparan sulfate. NPC1L1: NPC1 like intracellular cholesterol transporter 1. NTCP: Na^+^-taurocholate co-transporting polypeptide (SLC10A1). OCLN: occludin. SRBI: scavenger receptor class B type I.

## Data Availability

Not applicable.
